# Oncologists’ perceptions of tumor genomic profiling and barriers to communicating secondary hereditary risks to African American cancer patients

**DOI:** 10.1186/s12885-024-12184-y

**Published:** 2024-04-02

**Authors:** Michael J. Hall, Paul A. D’Avanzo, Yana Chertock, Patrick J. A. Kelly, Jesse Brajuha, Katie Singley, Caseem C. Luck, Sarah B. Bass

**Affiliations:** 1 Department of Clinical Genetics, Fox Chase Cancer Center, Philadelphia, PA USA; 2 College of Public Health, Department of Social and Behavioral Sciences, Temple University, Philadelphia, PA USA

**Keywords:** Oncology, Tumor genomic profiling, African American population, Genetic testing, Health disparities, Hereditary cancer risk, Health literacy, Barriers to healthcare, Cancer burden

## Abstract

**Background:**

Tumor genomic profiling (TGP) identifies targets for precision cancer treatments, but also secondary hereditary risks. Oncologists are poorly trained to communicate the results of TGP, especially among patients with lower health literacy, poorer genetics knowledge, and higher mistrust. African American (AA) patients are especially vulnerable to poor understanding due to significant cancer disparities and lower uptake of TGP. The goal of this research is to inform the development of an internet-based brief educational support for oncologists to prepare them to provide better decisional support related to TGP for their AA cancer patients.

**Methods:**

This mixed-methods study used semi-structured interviews of oncologists to inform development of an online survey with a convenience sample of US-based oncologists (*n* = 50) to assess perceptions of the challenges of TGP and communicating results to AA patients.

**Results:**

Most interviewed oncologists felt it was important to consider racial/cultural differences when communicating about hereditary risks. Cost, family dynamics, discrimination concerns, and medical mistrust were identified as particularly salient. Survey respondents’ views related to AAs and perceptions of TGP were strongly associated with years since completing training, with recent graduates expressing stronger agreement with statements identifying barriers/disadvantages to TGP for AA patients.

**Conclusions:**

Oncologists who had more recently completed training expressed more negative perceptions of TGP and more perceived challenges in communicating about TGP with their AA patients. Focused training for oncologists that addresses barriers specific to AAs may be helpful in supporting improved communication about TGP and improved decisional support for AA patients with cancer considering TGP to evaluate their tumors.

**Supplementary Information:**

The online version contains supplementary material available at 10.1186/s12885-024-12184-y.

## Background

The use of multi-gene tumor genomic profiling (TGP) to examine genes in a patient’s tumor for targetable mutations has become a cornerstone of modern personalized oncology [1, 2]. TGP is a DNA-based test of the tumor genome that uses next-generation sequencing to identify tumor-specific mutations and other genetic events that may be targetable for therapy. A 2017 survey of oncology providers found that over 75% had used TGP in their clinical practice to guide treatment decisions [3, 4]. As the power and accuracy of TGP has grown, costs have declined, enabling growing access to this critical diagnostic technology [5]. To identify tumor-specific mutations eligible for targeted therapy, TGP compares tumor DNA to germline DNA either from the same patient and/or by way of private or publicly available genomic databases. As a result, TGP may secondarily identify inherited mutations in cancer risk genes such as *BRCA1/2* and others. Multiple studies have found that actionable secondary germline mutations identified by TGP are prevalent in cancer patients, with 10–15% of patients undergoing TGP having a pathogenic or likely pathogenic mutation in an actionable hereditary cancer risk gene [6, 7]. These findings have significant implications for patients, and yet the potential for their discovery in the course of evaluating patients for cancer treatment utilizing TGP can complicate physician-patient communication about TGP risks and benefits [8–10].

African American (AA) patients face greater barriers to healthcare access and equitable treatment, resulting in higher cancer burden [11] and poorer outcomes across many cancer sites [12, 13], including higher rates of colorectal cancer, prostate cancer, lung cancer, hepatobiliary cancer, stomach cancer, and myeloma, among others [11, 14]. AA men and women also experience higher mortality from cancer overall [15]. Where hereditary/germline genetic testing is considered, numerous studies have also identified barriers to equitable care in the AA population, including reduced access to genetic services, poorer understanding and knowledge about genetics, and higher concerns about social stigmatization and healthcare or employment discrimination [16–21]. With the growing importance of TGP in guiding cancer treatment, disparities in genetic testing in the AA population are increasingly relevant to the oncology clinic. Notably, AA patients may be particularly vulnerable to care disruptions related to TGP due to cost concerns, poor understanding of TGP and its implications, and decision-making needs around management of hereditary risks [19, 20].

The American Society of Clinical Oncology (ASCO) has mandated that oncologists communicate risks of secondary germline findings from TGP to patients, and that patients’ preferences for managing results be considered and respected [22]. This includes the choice of opting out of receiving the germline information, having the doctor participate in conversations with family members, or referring for genetic counseling for additional information and support. However, oncology providers face several challenges in their efforts to communicate about TGP and secondary germline risks to patients, to assess patient preferences for management of personal genetic information, and to guide genetic risk decision making effectively, especially in more vulnerable populations like AA cancer patients [17–21, 23–32]. Indeed, few resources exist to support oncologists in these discussions, receiving little to no formal training in the interpretation and communication of potential secondary germline risks [33–36] or in supporting patients in decision making related to genetic information and risks. These factors increase the likelihood that patients undergoing TGP may be inadequately informed about secondary genetic findings or may receive information they did not want to receive. Despite this, little research has been conducted among oncologists to investigate the potential perceived barriers to TGP use in the clinic and TGP-associated communication. Studies by Hamilton have examined patient perceptions of hereditary risks from TGP, but these studies focused on qualitative research and the patient perspective [8–10]. Ademuyiwa et al. have surveyed clinicians about how patient perceive of genetics, but these authors did not examine TGP, nor did they seek to identify predictors among providers that may underlie variations in perceptions and behaviors [37].

The overarching study within which the current research falls is called E-IMPART. E-IMPART is a multi-level study funded by the American Cancer Society whose goal is to develop decisional supports for AA cancer patients and oncologists to promote better discussions and more informed decision-making for AA patients related to TGP and management of secondary hereditary results. The current study conducted formative work using a mixed-methods approach to explore oncologists’ perceptions and experiences with TGP. This is an appropriate method to use when little is known about a topic; combining qualitative and quantitative methods allows for broadening the understanding of a topic and informing intervention development [38]. Thus, the aim was to provide foundational information on how best to improve informed decision making for AA patients undergoing TGP by creating interventions for both patients and providers. Findings were used in the development of a short internet-based intervention for oncologists to expand their understanding and perceptions of the challenges of communication and decision support for AA cancer patients. We also explored how oncologists’ subspecialty, the recency of their training in oncology, and their technical knowledge of genetics and TGP, impacted their perceptions of TGP and use of TGP in the outpatient clinic to inform content of the intervention.

## Methods

### Overall study design

A mixed-methods research design was used, a common method in formative research in an area where little is known about a topic [39]. Oncologists’ perceptions of TGP-associated communication were assessed in two ways: first, a sample of practicing oncologists was recruited to complete semi-structured, one-on-one interviews in person or by telephone (*n* = 10). These interviews informed the development of an online survey that was developed by the study team and administered to a nationwide convenience sample of practicing oncologists (*N* = 50) who had recently received a TGP result from a commercial laboratory. Eligibility criteria for the interviews and survey were that participants were practicing oncologists who had recently ordered TGP through a commercial TGP provider. For the interviews, oncologists were from the greater Philadelphia, PA area. For the survey, oncologists who had ordered a TGP test through CARIS Life Sciences received an invitation to participate. If they were interested, they clicked on a link to the survey. The study was reviewed and approved by the Fox Chase Cancer Center Institutional Review Board (IRB# 18-8006).

### Population and study procedures

#### Semi-structured interviews

A 22-item interview guide was developed based on an extensive literature search and informed by the expertise of the primary study team. Subject areas related to TGP use and TGP-associated communication queried during the interview included: familiarity with TGP and practice patterns with using TGP; perceived advantages and disadvantages of TGP in cancer care; and content, frequency, and timing of discussions of TGP with patients including secondary hereditary risks. TGP and genomic testing barriers in the AA cancer population, such as socioeconomic barriers and low health literacy, medical mistrust, non-compliance with medical advice, and fears of discrimination were also examined. The full interview guide is available in the Supplementary Material.

Oncologists from four cancer centers in the Philadelphia region were invited to take part in the study via recruitment letter and/or by email. Participants included oncologists practicing in an academic suburban tertiary cancer center as well as from three urban cancer practices serving a diverse population of privately insured, Medicaid, and uninsured patients. Interviews were conducted by study team members in the physician’s private office or by telephone between 10/2018–1/2019. Interviews were audio recorded, and participants were offered a gift card incentive as a thank-you for their time.

A qualitative and descriptive approach as described by Willis [40] was used to examine physicians’ perceptions of TGP, TGP-associated communication about secondary hereditary results, and barriers specific to AA patients. This qualitative approach is not intended to test or generate theory. Instead, it is a common method to gather explicit information about views and experiences when seeking to understand the implications of phenomena for healthcare practice or policy [41, 42] and is considered appropriate when exploring new areas of research where little information is available. A total of 10 interviews were conducted. Initial analysis showed that saturation had occurred, with specific themes emerging across the 10 interviews. Once saturation has been met, no further interviews are needed in qualitative formative work that is meant to provide information on a topic and not be generalized to a larger population or to test hypotheses [43].

#### Quantitative survey

Qualitative results guided development of the 84-item survey. Triangulation of data was achieved by having multiple interviewers and a coding scheme that ensured input from the study team. All transcripts were analyzed with at least two coders to reduce the risk of observer bias [44]. Nine areas relevant to provider perceptions of and attitudes toward TGP, such as providers’ TGP beliefs, providers’ own perceived benefits and disadvantages to TGP, and beliefs about how patients perceive TGP, were explored. For the current analysis, 25 items specific to the care of AA cancer patients were examined. These 25 items queried providers’ perceptions and beliefs about caring for AA cancer patients related to genetics, providers’ beliefs about how AA patients perceive TGP, communication about TGP, and beliefs about training needs related to TGP among peers practicing in the field of oncology.

Attitudes and perceptions of TGP were assessed on 11-point scales (0 to 10) measuring disagreement (0 = totally disagree) to agreement (10 = totally agree) with 28 statements about AA patients and/or TGP (e.g., “My African American patients are more likely to decline TGP testing thinking that this is something extra and not part of their treatment”). The survey also queried provider demographics, practice characteristics, years in clinical practice (i.e., recency of training completion) and volume of TGP use. Knowledge of TGP was assessed by a 7-item measure modified from our previous research [33]. For analyses, oncologists were secondarily subdivided by specialty (medical oncology *n* = 39 vs other oncology *n* = 11), experience or years in practice (≤ 10 years *n* = 11; 11–20 years *n* = 25; 21+ years *n* = 14), by knowledge level (low knowledge of genetics and TGP *n* = 24, high knowledge *n* = 26) and by the volume of testing conducted (< 50 tests/year *n* = 28; ≥ 50 tests/year *n* = 22).

In collaboration with Caris Life Sciences, a commercial laboratory that conducts TGP, invitation emails containing the study details, informed consent document, and survey link for the study were sent to providers who had recently ordered TGP. Participants who completed the survey were offered a gift-card incentive as a thank-you for their time. A total of 50 oncologists nationwide completed the online survey (11/2019–1/2020).

### Analysis

Completed qualitative interviews were transcribed and coded. To ensure confidentiality, all transcripts were de-identified. Fifteen initial codes were developed based on the interview guide; 80 additional codes were added during the analysis as further themes emerged. Two team members met during the coding process to reach consensus, update the coding structure, and refine previously coded text. A priori codes drawn from the interview guide served as the organizing framework for analyses. Following iterative analysis of all transcripts, the finalized draft of the codebook included 6 core overarching themes: knowledge of genomics; advantages and disadvantages of TGP; discussing TGP with patients; value and harm of TGP to patients; unique risks of TGP to AA patients; and need for additional training for providers who use TGP.

Quantitative surveys were analyzed by SPSS [IBM Corp. Released 2017. IBM SPSS Statistics for Windows, Version 25.0]. Demographic and practice characteristic data were described using means (continuous variables) and frequencies (categorical variables). Differences in attitudes and perceptions of TGP by provider subgroup were tested using two-sided, two-sample t-tests (α = 0.05) and ANOVA to evaluate mean differences between or among groups on the survey items. No adjustments were made for multiple comparisons. The figure was created using R 4.3.0 (R Core Team, 2023).

## Results

### Semi-structured interviews

Ten oncologists of diverse sex, race/ethnicity, and years in practice were interviewed (see Table 1). Interviews identified several dominant concepts associated with perceived TGP risks and challenges in results communication specific to vulnerable patient populations such as AA patients, including: socio-economic barriers to TGP; mistrust of genetics, research, clinical trials and more generally mistrust of health care overall; social suspicion and privacy; fears of discrimination and stigmatization; and non-compliance with medical care. A selection of relevant quotes related to use of TGP in AA patients is provided in Table 2.

**Table 1:** Characteristics of oncologists participating in semi-structured interviews and completing online survey

**Characteristics**	**Semi-structured interviews (*n* = 10)** **N (%)**	**Online survey of oncologists who ordered TGP (*n* = 50)** **N (%)**
**Specialty**		
Medical Oncology	10 (100)	39 (78)
Surgical Oncology		4 (8)
Gynecologic Oncology		7 (14)
**Practice type**		
Academic center based practice	10 (100)	35 (70)
Academic center affiliated private practice		3 (6)
Community hospital practice		10 (20)
Private practice		2 (4)
**Gender**		
Male	4 (40)	33 (66)
Female	6 (60)	15 (30)
N/A		2 (4)
**Race/Ethnicity**		
Black/African American	4 (40)	3 (6)
Asian	3 (30)	18 (36)
Latina/o/x		3 (6)
White	3 (30)	26 (52)
**Age**		
Mean	41 years	45 years
**Clinical Experience**		
≤ 10 years	7 (70)	11 (22)
11–20 years	3 (30)	25 (50)
≥ 21 years	0	14 (28)
**TGP testing volume**		
Mean	NA	50 tests/year
25th percentile	NA	30 tests/year
75th percentile	NA	100 tests/year
Range	NA	2–400 tests/year

**Table 2:** Select quotes from semi-structured interviews (*n* = 10)

**Participant**	**Special considerations related to genetics and use of TGP in AA cancer patients due to concerns about discrimination, high mistrust, and poor understanding**
**Discrimination concerns**	
ONC7	*I think doctors need to take these [into] consideration when talking to underserved [patients], not only in terms of race, but also socio-economic status as well, because many times people who have poor socio-economic status they think they get lesser care and when you offer these things that they don’t understand they might see it as a way to discriminate against them. So, explaining to them, “This is a standard thing and I offer it to everyone, and it is how I may help you.”*
**Medical mistrust**	
ONC3	*I think emotional risk probably is pretty high because of the mistrust. [Patients] go to [their] wife/husband and say they [doctors] want to do genetic testing on me and they [family] would say “don’t do it”, because this is the way [they do things] to not treat you, this is the way of finding something that will harm you, instead of help you.”*
ONC8	*My biggest push back from AA patients more than other races, is that they don’t like the idea of experimental types of care. Some patients if you mention [the] words “clinical trial” they’re really not…aligned with the term “clinical trial”. They consider it as something experimental, ‘it doesn’t benefit me’, and ‘I’m not a guinea pig’ — these are the kind of [remarks] I’ve heard a lot*
**Poor awareness/understanding of genetics**	
ONC8	*I’ve noticed it being a biggest difference when I’m talking about things like tumor profiling; sometimes it’s [due to] a bigger educational gap, so you really have [to make] that dedicated effort to explain what TGP is, very clearly*
ONC5	*To middle- aged and older people it’s hard to sell genomic profiling. They just don’t want to understand. They say, “I’ll do whatever you need, where do I sign?” “It’s just too much, I don’t understand what are you saying.” “Do what you have to do.”*
**Uniform vs patient-tailored approach to TGP communication**	
ONC4	*I think you should approach people the same way, and yet every patient needs something different from you. And there are maybe specific things to address but….[pause]. I think that these tests are not well understood by patients across the board. I don’t think it’s necessarily specifically racial differences that I have seen*
ONC6	*I think in general I present options and information the same way. I think AAs sometimes, depending on educational level [need more explanation], but anybody … with low health literacy or lower SES [needs this too], and so you need to able to explain it clearly, issues like cost maybe more often. I treat prostate cancer, and certainly … AAs maybe [have] higher risk of mutations, but I’m not sure I will present it somehow differently*
ONC10	*A generic approach at discussion works for many but not for all [patients]. Obviously, we want to take into consideration cultural sensitivities, cultural appreciation, racial and socio- economic boundaries. I think as a clinician one has to be sensitive… in general*

Several oncologists, particularly those whose practices included more unban underserved AA patients, identified limited health literacy and limited knowledge of genetics as important barriers to informed health decisions among many of their AA patients. They agreed that the conscious effort to use plain language when talking about complex topics such as TGP, and setting aside extra time to thoroughly explain all aspects of testing and answer questions, are often effective at supporting improved comprehension.

Medical mistrust was also a salient feature of interactions with AA cancer patients and was described as having a negative impact on genetic testing and the communication of genetic findings. Discussions of TGP were noted to be more difficult if the purpose of performing TGP testing was specifically connected to clinical trial eligibility, due to underlying negative attitudes towards clinical trials among AA patients. Several oncologists noted that their AA patients are often warier of genetic tests than White patients and have concerns that testing is experimental. Oncologists also shared that their AA patients sometimes have suspicions that TGP testing is just being done to make money off their insurance or for secondary gain on the part of the oncologist or the cancer center. Strong rapport building between the provider and the patient was suggested as the most powerful way to acknowledge and navigate medical mistrust concerns. Illustrating this, one respondent said: *“There is a lack of trust of the healthcare system, and ...that there is more personal gain to be had [by] the healthcare provider rather than the primary driving force being for the good of the patient. I have some patients from [the] AA population…who say: ‘Is this because I have good insurance? Is this because you are paid more if you do this?’ I think the level of suspicion and hesitancy exists in this patient population, and it’s different from other patient populations.” (ONC3).*

Oncologists similarly noted that their AA patients more often worry about discrimination specifically rooted in genetics. They described that conversations about TGP and hereditary risks could raise alarm and evoke negative reactions. Oncologists suspected this may also lead to patients not following through with genetic evaluation. One respondent said, *“Minorities do get exploited more. They worry that something like this will be another excuse for insurance [companies] to deny them, or to not get something approved. Because this happens all the time for different reasons. The whole denial thing ‘I’m gonna deny you this, you’re not gonna get this’— they hear it all the time. If there is any reason they should fear that this test [TGP] is going to [lead to denials of care] maybe not [for] them but their children or grandchildren, that really scares them… And we [physicians] don’t have enough knowledge to tell them ‘don’t be afraid’ or ‘don’t worry’” (ONC5).*

Oncologists were divided on whether special considerations based on race/ethnicity should be made when formulating discussions of TGP and management of hereditary cancer risk information. Three participants did not favor actively taking race or ethnicity-specific beliefs and barriers into consideration. Conversely, several others strongly supported the idea that oncologists need to consciously recognize important factors such as racial and cultural sensitivity, socio-economic differences, and medical mistrust when having these discussions. One respondent said, *“Yes, you want to treat patients the same by offering the same treatment, but at the same time I think understanding that, especially when [an] African American patient says ‘I don’t want to do it’, you really need to ask why not, because it really can go back to these historical aspects where they’re afraid they will be discriminated against in some way” (ONC7)*. Finally, oncologists similarly had mixed opinions as to whether they should directly address some of the barriers specific to their AA patients when leading discussions. Several providers agreed that it is important to address barriers with patients. However, others were less confident about the nature and content of these discussions.

### Survey results

Characteristics of oncologists completing the online survey are seen in Table 1. Participant oncologists were 66% male with a mean age of 45 years. Most were medical oncologists (78%) with a mean of 17.7 years in practice (range: 7–39 years). The mean number of TGP tests sent annually was 50 [range 2–400]. The median score on the knowledge measure was 5 [range 3–7] with 48% of oncologists scoring ≤ 5.

#### Concerns and barriers to TGP

**Fig. 1:**
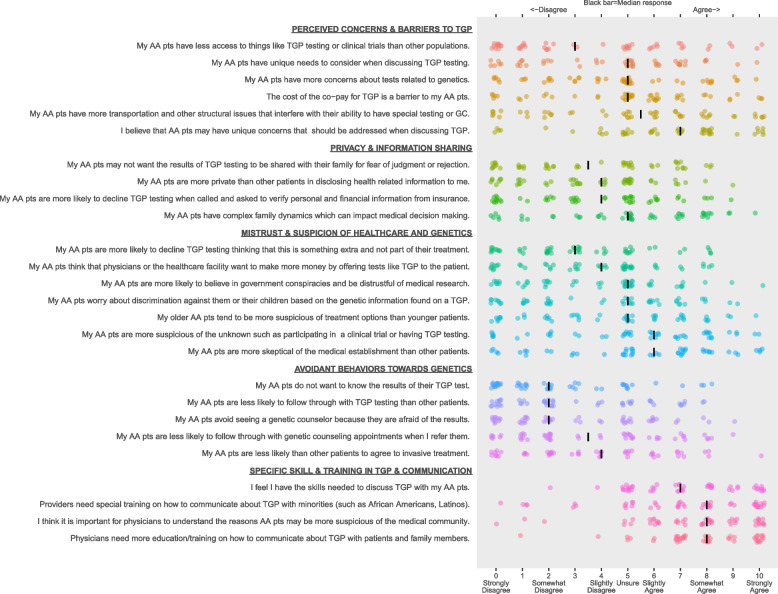
Oncologists’ perceptions of tumor genomic profiling in African American cancer patients. For each survey item, the colored dots depict each participant’s response; these dots have been jittered around the response value to limit overplotting. The vertical bar indicates the median response

Results on statements regarding TGP and AA patients are presented in Supplemental Table 1 and Fig. 1. On items related to specific concerns or potential barriers to TGP testing in AA patients, in stratified analyses two items demonstrated significant differences: medical oncologists more strongly agreed with access barriers to TGP and clinical trials for AA patients than did non-medical oncologists (5.05 vs 3.09, *p* = 0.029), while oncologists who reported lower overall use of TGP in their practices agreed more strongly to the unique concerns AA patients may have related to TGP that should be addressed (mean 7.93 vs 6.36, *p* = 0.042).

#### Privacy and health information sharing

Four items examined privacy related to health information disclosure to third parties (e.g., healthcare organizations) and medical privacy relevant to family members, where family dynamics and concerns about judgment and blame related to genetics may be heightened. In stratified analyses, less experienced oncologists (those in practice ≤ 10 years) agreed more strongly with the relevance of overall health information privacy (mean 6.64, SD 1.03) compared with more experienced providers (11–20 years’ experience: mean 3.88, SD 2.21; 21+ years’ experience: mean 4.29, SD 6.27; *p* = 0.003) and privacy related to TGP-related personal and financial information verification (mean 5.82, SD 2.40) than more experienced providers (11–20 years: mean 4.04, SD 2.32; 21+ years: mean 3.21, SD 2.16. *p* = 0.040). Similarly, oncologists overall did not believe that AA patients would refrain from sharing TGP results with family due to concerns of judgment or rejection (mean 4.62, SD 2.85), although stratified analyses showed that early career oncologists were more likely to endorse this notion (≤ 10 years: mean 6.64, SD 1.63; 11–20 years: mean 3.84, SD 2.69; 21+ years: mean 4.43, SD 2.82, *p* = 0.014). While participants tended to agree that AA patients have complex family dynamics that can impact decision making (mean 6.00, SD 2.72), stratified analyses were not significant.

#### Medical mistrust

Six items examined oncologists’ perceptions of mistrust toward medicine and genetic/genomic testing among their AA patients. In stratified analyses, oncologists with less practice experience agreed more with the statement, “My AA patients are more likely to decline TGP testing thinking this is something extra and not part of their treatment” (≤ 10 years: mean 5.82, SD 2.40; 11–20 years: mean 4.04, SD 2.32; 21+ years: mean 3.21, SD 2.16, *p* = 0.023).

#### Patient behaviors

Oncologists’ perceptions of behaviors of AAs relative to genetics and TGP were queried in 5 items, and 4 showed significant differences in perceptions of AA when stratified by years of experience in practice. Oncologists with less practice experience (≤ 10 years) agreed more strongly that “My AA patients are less likely than other patients to agree to invasive treatments” [mean 7.09, SD 1.92 vs 4.04, SD 2.46 (11–20 years) and 4.00, SD 2.60 (21+ years)(*p* = 0.002)], “My AA patients do not want to know the results of their TGP test” with means of 5.00, SD 2.05 (≤ 10 years), 3.24, SD 2.49 (11–20 years) and 2.79, SD 2.12 (21+ years) (*p* = 0.05)], and “My AA patients are less likely to follow through with TGP testing than other patients” with means of 5.73, SD 2.33 (≤ 10 years), 3.76, SD 2.65 (11–20 years) and 3.36, SD 2.65 (21+ years)(*p* = 0.05). Finally, oncologists believed that AA patients may also avoid support from a genetic counselor: “My AA patients avoid seeing a genetic counselor because they are afraid of the results” with means of 5.82, SD 2.40 (≤ 10 years) 3.80, SD 2.66 (11–20 years) and 3.36, SD 2.24 (21+ years) (*p* = 0.042). Overall, less experienced oncologists consistently agreed more strongly that their AA patients exhibited avoidant behaviors toward genetics and genetic testing.

#### Providers perceptions of skills and need for additional training

The 4 final items assessed oncologists’ perceptions of their skills in communicating about TGP with their AA patients, the importance of understanding barriers to TGP among AAs, and the need for more training in this discipline. When comparing by overall knowledge level about TGP, oncologists had confidence in their own skillset in this regard [“I feel I have the skills needed to discuss TGP with my AA patients” (mean 8.16, SD 1.99)] but they agreed strongly, especially those with lower knowledge, that more training is needed [“Physicians need more education/training on how to communicate about TGP with patients and family members” (low-knowledge mean 9.54, SD 1.41, high knowledge mean 8.23, SD 2.03, *p* = 0.011)].

## Discussion

Diagnostic genomic testing, including TGP, is increasingly critical to cancer prognosis and cancer treatment planning, yet vulnerable populations like AA patients are at risk of poorer understanding, poorer decision-making, and poorer outcomes from genomic testing [45]. Research focused on understanding individual-patient– and sociocultural differences among AA cancer patients and other vulnerable racial/ethnic groups is needed to anticipate and address barriers to clinical trials and genomic testing in underserved populations and to mitigate treatment and outcome disparities that may result [33, 46, 47]. The central role of TGP in cancer-treatment planning has grown considerably in just a few years; while TGP is still largely used in a setting of more advanced disease, where targeted therapies are more prevalent, comprehensive molecular assessment via TGP is likely to become more common in earlier cancer stages as additional relevant molecular markers of prognosis and treatment are identified [2, 5]. No studies to our knowledge have specifically examined the challenge of communicating secondary hereditary results from TGP to patients *from the provider perspective* and *in an underserved population*. Studies by Gray et al. and others have examined perceptions and challenges of communicating TGP somatic findings to patients [48–50], but few have explored germline risks other than those studies determining the prevalence of germline hereditary risks in patients undergoing TGP [6, 7]. Research by Hamilton et al. examining perceptions of hereditary risks of TGP [8–10] has examined patient perceptions of tumor genomic profiling or somatic testing of cancers, but has been focused on the patient perspective and in a largely White patient sample, while that of Ademuyiwa [37] focuses on oncologists, but is limited to studying perceptions of hereditary risk testing. Our research therefore builds and expands on the findings of these authors to identify communication and information barriers and needs from both the patient and provider perspective. This study specifically focuses on formative research to support development of a brief online educational intervention for oncology providers.

In the current study, we asked oncologists focused items which probed perceived barriers and challenges specific to AA cancer patients and stratified our survey results for our analyses of perceptions by factors that could be relevant in distinguishing attitudinal variation among providers. Interviews produced a rich variety of perceptions about the challenges of using TGP in practice with AA cancer patients, including the perception of poorer understanding of genetics among AA patients, experiencing more negative views of genetics rooted in historical abuses of AAs in medical research, and mistrust of physicians and genomics. In our survey of oncologists, we found that oncologists perceived barriers to TGP and genetics in their AA population. Notably, across items querying perceptions related to access, privacy, mistrust, and avoidance, significant variability in perceptions by years of experience in practice was seen, with younger, less-experienced oncologists tending to have stronger agreement with statements identifying barriers. Oncologists overall felt efforts to increase recognition of barriers specific to AA patients was important. Furthermore, oncologists felt more training and education are needed in TGP communication - despite significant growth in the indications for TGP in the assessment and treatment of diverse tumors, and the significant increase in the complexity of the testing being conducted and reported (e.g., numbers of genes, types of testing, bio-informatic predictors), providers receive minimal training in basic genetic-risk communication, not to mention the communication of complex genetic findings. There is clearly a need for ongoing, dynamic education for oncologists in the realm of tumor genomics and risk communication, and our study is among the first to focus on this gap in the AA cancer population.

The central challenge of TGP is the potential to receive at least two relevant pieces of information in one complex genetic test: one result that guides treatment through analysis of tumor specific somatic DNA targets, and a second result that catalogues germline or inherited DNA mutations, the identification of which both increases the power of the somatic testing to detect treatment relevant mutations but also reveals sensitive secondary information on hereditary cancer risk. Complex, multi-level information is not commonly encountered with most medical tests, and thus the perceptions of patients toward secondary hereditary information, and the extent to which patients may require additional preparation to receive it, has been minimally studied. Importantly, the majority of studies that have looked at perceptions of TGP more broadly have not focused specifically on AA or other racial/ethnic minorities, with most reporting fewer than 15% of the sample as non-White [51–54]. The research of Hamilton et al. [8–10], which has focused specifically on patient perceptions of secondary hereditary findings of TGP, has also not focused on the perceptions of more vulnerable populations. In a qualitative study with a sample of 40, only one AA patient is noted [8–10].

Generally, TGP decisions are driven first and foremost by the desire to find new therapies and the desire to survive a life-threatening disease. Many patients are equivocal or disinterested in secondary hereditary findings, and most believe they should be able to refuse this information. Patients with advanced cancer are likely to value the secondary hereditary information less than their providers do and to have greater concerns about emotional harms and information control, highlighting the importance of patients being fully informed about secondary hereditary findings to support testing decisions, including the decision to opt-out of receiving this information. While federal law (GINA) protects individuals with hereditary risks from discriminatory practices related to health insurance premiums, fears of insurance discrimination are commonly reported by patients, and the GINA laws do not protect against life insurance and long-term care insurance. But importantly, the lack of representation of patients of color in previous work creates a gap in our understanding of how psychological factors like medical mistrust and fears of discrimination add to or retract from reactions to getting secondary germline findings, and how this ultimately impacts communication between providers and patients [55]. This study is one of the first to understand how provider beliefs may impact that communication.

Our study is also unique in its stratification of oncologists’ perceptions of barriers to TGP testing in AA patients, and lends insight to the relationship of subspecialty, genetics knowledge, testing familiarity, and recency of medical training to perceptions of genetic medicine and how this is influenced by patient race. We observed that oncologists earlier in their career agreed more strongly with negative beliefs related to how their AA patients view TGP. Ademuyiwa et al. also queried US-based breast oncology providers perceptions of how AA patients with breast cancer experience genetic testing [37]. Similar to our findings, oncologists generally reported more barriers to genetic counseling among their AA patients, such as non-compliance and mistrust, compared to White patients, and expressed concerns that AA patients are more likely to get ambiguous results, and to require decisional support. Overall, these results and our own lend support to the need for decision-making supports in the AA cancer patient population undergoing genetic tests like TGP, and among oncologists taking care of these patients. One important limitation of Ademuyiwa et al. is that they did not stratify results by provider characteristics such as knowledge or years since completing training. Our findings show that more experienced providers largely perceive fewer barriers to use of TGP in AA patients, while less experienced (more recently trained) providers perceive more; this may reflect both a greater caution and awareness among more recently trained providers toward genetics and greater recognition of barriers unique to minority and underserved populations, or perhaps greater indifference to barriers among more experienced providers.

Limitations of our research include the relatively small sample sizes for the semi-structured interviews and the survey, and our decision to focus only on patients of AA ancestry. Nonetheless, we have identified strong and relevant themes with these samples that have guided our development of a patient-focused decision support tool and an oncologist-focused educational tool. In addition, ongoing research from our group expands our research to patients of Hispanic/Latinx ancestry in an effort to begin to understand perception and barriers to genomics and hereditary-risk information in this population.

## Conclusion

- The increased use of tumor genomic profiling (TGP) in the oncology clinic places vulnerable patients like AAs at risk of having testing conducted with limited awareness and understanding.
- Oncologists recognize their limited skills and poor preparation to communicate risks, benefits and results of TGP to their AA patients, especially secondary hereditary risks.
- Results of this formative research that identifies oncologists’ perceptions of TGP use in their AA patients will inform an online educational intervention for oncologists to prepare them to have better and more informed conversations about TGP with their AA patients.

### Supplementary Information

**Supplementary Material 1.**

## Data Availability

The datasets used and/or analyzed during the current study are available from the corresponding author on reasonable request.

## Abbreviations

AA: African American
TGP: Tumor genomic profiling
ASCO: American Society of Clinical Oncology
