# Supplementary material for: Oncologists’ perceptions of tumor genomic profiling and barriers to communicating secondary hereditary risks to African American cancer patients
Source: BMC Cancer. 2024 Apr 2;24:412. doi: 10.1186/s12885-024-12184-y (PMC10988900; doi:10.1186/s12885-024-12184-y)
Supplement: Supplementary file 1 — Supplementary Material 1. [file 12885_2024_12184_MOESM1_ESM.docx]

**Supplemental Table 1: Oncologists’ perceptions of TGP use in AA cancer patients, stratified by specialty, years of experience in practice, knowledge, and volume of testing**

|  | **Specialty Med (39) vs other (11)** | **Experience**  **<10 n=11**  **11-20 n=25**  **21+ n=14** | **Knowledge**  **Low n=24**  **High n=26** | **Volume testing**  **<50 n=28**  **>50 n=22** |
| --- | --- | --- | --- | --- |
| My African American patients are more skeptical of the medical establishment than other patients | 6.62  5.91      P=0.428 | 7.27  6.28  6.14    P=0.589 | 6.54  6.38      P=0.853 | 6.14  6.86      P=0.407 |
| My African American patients are more suspicious of the unknown such as participating in a clinical trial or having TGP testing | 6.41  5.91    P=0.573 | 6.91  6.44  5.57  P=0.489 | 5.96  6.62    P=0.425 | 6.18  6.45    P=0.746 |
| My African American patients have more concerns about tests related to genetics | 5.59  5.18    P=0.639 | 6.45  5.40  4.93  P=0.378 | 5.42  5.58    P=0.840 | 5.68  5.27    P=0.616 |
| My African American patients have complex family dynamics which can impact medical decision making | 6.00  6.00    P=1.0 | 7.18  5.20  6.50  P=0.094 | 6.58  5.46    P=0.150 | 5.75  6.32    P=0.474 |
| My African American patients are less likely than other patients to agree to invasive treatment | 4.77  4.45    P=0.732 | **7.09**  **4.04**  **4.00**  **P=0.002** | 4.83  4.58  P=0.740 | 4.50  4.95  P=0.567 |
| My African American patients think that physicians or the healthcare facility want to make more money by offering tests like TGP to the patient | 4.82  4.27    P=0.428 | 5.64  4.52  4.29  P=0.337 | 4.46  4.92    p=0.503 | 4.71  4.68    P=0.964 |
| My African American patients are more private than other patients in disclosing health related information to me | 4.59  4.64    P=0.945 | **6.64**  **3.88**  **4.29**  **P=0.003** | 4.63  4.58    P=0.944 | 4.71  4.45    P=0.710 |
| My older African American patients tend to be more suspicious of treatment options than younger patients | 5.62  4.45    P=0.164 | 4.60  6.55  5.08  P=0.291 | 5.29  5.42    P=0.872 | 5.18  5.59    P=0.633 |
| My African American patients are more likely to believe in government conspiracies and be distrustful of medical research | 4.90  4.82    P=0.924 | 5.73  4.64  4.64  P=0.486 | 4.96  4.81    P=0.842 | 4.96  4.77    P=0.801 |
| My African American patients are more likely to decline TGP testing when called and asked to verify personal and financial information from insurance | 4.85  4.27    P=0.490 | **6.36**  **4.52**  **3.79**  **P=0.040** | 4.79  4.65    P=0.855 | 4.93  4.45    P=0.545 |
| My African American patients are more likely to decline TGP testing thinking that this is something extra and not part of their treatment | 4.26  4.40    P=0.733 | **5.82**  **4.04**  **3.21**  **P=0.023** | 4.25  4.15    P=0.891 | 4.46  3.86    P=0.408 |
| My African American patients have unique needs to consider when discussing TGP testing | 5.33  5.27    P=0.943 | 5.82  5.20  5.14  P=0.801 | 5.58  5.08    P=0.525 | 5.64  4.91    P=0.360 |
| My African American patients do not want to know the results of their TGP test | 3.56  3.27    P=0.693 | 5.00  3.24  2.79  P=0.051 | 3.54  3.46    P=0.908 | 3.57  3.42    P=0.820 |
| My African American patients are less likely to follow through with TGP testing than other patients | 4.21  3.64    P=0.519 | **5.73**  **3.76**  **3.36**  **P=0.048** | 4.29  3.88    P=0.585 | 4.14  4.00    P=0.853 |
| My African American patients are less likely to follow through with genetic counseling appointments when I refer them | 4.92  4.00    P=0.274 | 6.18  4.44  4.07  P=0.115 | 4.75  4.69    P=0.941 | 4.54  4.95    P=0.609 |
| My African American patients have more transportation and other structural issues that interfere with their ability to have special testing or genetic counseling | 6.41  5.09    P=0.127 | 7.73  5.80  5.43  P=0.155 | 6.00  6.23    P=0.800 | 5.93  6.36    0.638 |
| My African American patients may not want the results of TGP testing to be shared with their family for fear of judgment or rejection | 4.72  4.27    P=0.621 | **6.64**  **3.84**  **4.43**  **P=0.014** | 4.83  4.42    P=0.599 | 5.04  4.09    P=0.235 |
| My African American patients worry about discrimination against them or their children based on the genetic information found on a TGP | 5.18  4.09    P=0.247 | 6.09  4.72  4.43  P=0.309 | 5.60  4.42    P=0.186 | 5.07  4.77    P=0.718 |
| My African American patients have less access to things like TGP testing or clinical trials than other populations | **5.05**  **3.09**    **P=0.029** | 5.27  4.40  4.50  P=0.738 | 5.25  4.04    P=0.170 | 4.46  4.82    P=0.697 |
| My African American patients avoid seeing a genetic counselor because they are afraid of the results | 4.26  3.64    P=0.457 | **5.82**  **3.80**  **3.36**  **P=0.042** | 4.38  3.88    P=0.515 | 4.14  4.09    P=0.947 |
| The cost of the co-pay for TGP is a barrier to my African American patients | 6.38  5.18    P=0.106 | 6.36  5.72  6.64  P=0.593 | 6.33  5.92    P=0.612 | 5.93  6.36    P=0.600 |
| I believe that African American patients may have unique concerns that should be addressed when discussing TGP | 7.49  6.36    P=0.175 | 8.09  7.00  7.00  P=0.502 | 7.33  7.15    P=0.815 | 7.93  6.36    P=0.042 |
| I feel I have the skills needed to discuss TGP with my African American patients | 8.33  7.55    P=0.360 | 8.36  8.20  7.93  P=0.860 | 7.67  8.62    P=0.097 | 7.93  8.45    P=0.249 |
| Physicians need more education/training on how to communicate about TGP with patients and family members | 8.95  8.55    P=0.567 | 9.00  8.48  9.43  P=0.306 | **9.54**  **8.23**    **P=0.011** | 8.68  9.09    P=0.432 |
| Providers need special training on how to communicate about TGP with minorities (such as African Americans, Latinos) | 8.18  7.36    P=0.399 | 7.82  7.44  9.14  P=0.169 | 8.58  7.46    P=0.146 | 7.96  8.05    P=0.918 |
| I think it is important for physicians to understand the reasons African American patients may be more suspicious of the medical community | 8.74  7.45    P=0.157 | 8.64  7.92  9.29  P=0.199 | 8.79  8.15    P=0.323 | 8.61  8.27    P=0.623 |
